# The MULTISENSE Test of Lexical–Gustatory Synaesthesia: An automated online diagnostic

**DOI:** 10.3758/s13428-019-01250-0

**Published:** 2019-06-03

**Authors:** Alberta Ipser, Jamie Ward, Julia Simner

**Affiliations:** School of Psychology, University of Sussex, Brighton, UK

**Keywords:** Lexical–gustatory synesthesia, Synesthesia, Automated consistency test, Taste

## Abstract

Lexical–gustatory (LG) synesthesia is an intriguing neurological condition in which individuals experience phantom tastes when hearing, speaking, reading, or thinking about words. For example, the word “society” might flood the mouth of an LG synesthete with the flavor of fried onion. The condition is usually verified in individuals by obtaining verbal descriptions of their word–flavor associations on more than one occasion, separated by several months. Their flavor associations are significantly more consistent over time than are those of controls (who are asked to invent associations by intuition and to recall them from memory). Although this test reliably dissociates synesthetes from nonsynesthetes, it suffers from practical and methodological limitations. Here we present a novel, automated, online consistency test, which can be administered in just 30 min in order to instantly and objectively verify LG synesthesia. We present data from two versions of our diagnostic test, in which synesthetes report their synesthetic flavors either from a hierarchical set of food categories (Exp. 1) or by specifying their basic component tastes (sweet, salty, bitter, etc.). We tested the largest sample of self-declared LG synesthetes studied to date and used receiver operating characteristic analysis to assess the discriminant power of our tests. Although both our methods discriminated synesthetes from controls, our second test (Exp. 2) has greater discriminatory power with a threshold cutoff. We suggest that our novel diagnostic for LG synesthesia has unprecedented benefits in its automated and objective scoring, its ease of use for participants and researchers, its short testing time, and its online platform.

**Electronic supplementary material:**

The online version of this article (10.3758/s13428-019-01250-0) contains supplementary material, which is available to authorized users.

Lexical–gustatory (LG) synesthesia is an intriguing neurological condition in which sounds induce phantom flavors (e.g., Ramachandra, 2016; Ward & Simner, 2003). People with LG synesthesia (known as *LG synesthetes*) experience floods of flavor in the mouth or intrusive food-related thoughts whenever they hear certain sounds, especially words. In some cases, people with LG synesthesia taste every single word they read, speak, hear, or even think about (e.g., Ward, Simner, & Auyeung, 2005). For example, when synesthete J.I.W. hears the word “audience,” his mouth is flooded with the flavor of tinned peas. The name “Phillip” fills his mouth with bitter oranges. And the word “society” tastes of onions (e.g., Ward & Simner, 2003). These flavors have been objectively verified in behavioral tasks (e.g., Ward & Simner, 2003; Ward et al., 2005) and tied to unusual neurological activity in the taste centers of synesthetes’ brains (e.g., the insula; Jones et al., 2011). LG synesthesia is just one of a number of different synesthesias recorded in the neuropsychological and medical literature, all of which cause unusual additional sensations and can affect multiple senses. For example, other synesthetes might “see” colored photisms in the visual field triggered by listening to music or reading (e.g., Dixon, Smilek, & Merikle, 2004; Ward, Huckstep, & Tsakanikos, 2006; see Simner & Hubbard, 2013, for a review).

Case reports (e.g., Gendle, 2007; Ramachandra, 2016; Richer, Beaufils, & Poirier, 2011; Ward & Simner, 2003) and small-group studies (e.g., Ward et al., 2005) have shown two ways in which LG synesthesia can be experienced. Synesthete J.I.W., for example, experiences LG synesthesia as if he were tasting veridical flavors in the mouth, with each word being like a droplet of taste on the tongue (Ward & Simner, 2003). In contrast, the flavors of synesthete S.K.M. are automatic and immediate “thought associations” between the inducing word and a food type (e.g., the word “dean” evokes the precise and consistent notion of minced beef in gravy, but nothing is tasted in the mouth). We will refer to these manifestations as “projector” and “associator” forms of LG synesthesia, respectively, taking these terms from related differences found in color-experiencing synesthetes (see Dixon et al., 2004). Whether the sensation is projected or associated, it is a complex food flavor (e.g., minced beef and gravy) rather than a pure taste (e.g., bitter) and can involve texture, temperature, and other multisensory components (e.g., “jail” tastes of cold hard bacon for synesthete J.I.W.; Ward & Simner, 2003). Finally, we point out that lexical–gustatory experiences can also include nonfoods such as synthetic materials (e.g., plastic), organic inedibles (e.g., earwax), and even abstract textures or shapes (e.g., something thin and rough; Richer et al., 2011; Ward & Simner, 2003).

Relatively little is known about LG synesthesia, although it is certainly extremely rare—the only attempt to verify its prevalence using an objective diagnostic test and wide-scale screening of the general population detected no cases at all within a sample of 500 people (Simner et al., 2006). This places the prevalence of LG synesthesia at less than 0.2%, although it may yet be rarer. One key problem is that there has never been a standardized way to diagnose LG synesthesia, and there is no available test that could be shared across researchers or clinicians. Our aim here is to present such a test: We describe two novel versions of a diagnostic tool for LG synesthesia and evaluate how effective each test is in distinguishing synesthetes from controls.

An objective test for LG synesthesia would be of key importance, because synesthesia cannot be reliably diagnosed by self-report alone. Even detailed questionnaires with clear information about the nature of synesthesia produce high rates of acquiescence bias in self-report measures, at least for some types of synesthesia (e.g., colored hearing; Simner et al., 2006). These false reports arise in part because synesthesia shares similarities with normal intuitive cross-sensory correspondences found in everyone; for example, all people are likely to associate “happiness” with, say, chocolate rather than spinach, or with the color yellow rather than black. Such similarities make it difficult for nonsynesthetes to confidently reject the notion of “synesthesia” or to understand the difference between normal associations and synesthetic ones. However, where this distinction can be objectively shown (see below), it predicts enormous differences in phenomenology (Ward & Simner, 2003), behavior (Simner & Logie, 2007), neurological activity (Jones et al., 2011), sensory sensitivity (Ward et al., 2017), and a range of other characteristics that separate synesthetes from nonsynesthetes. The aim of our research was therefore to produce a test of LG synesthesia to provide an objective means of diagnosis. We present two versions of our test below, and evaluate their effectiveness in distinguishing synesthetes from nonsynesthetes.

We developed our test from a consideration of previous methods. Participants have been validated as genuine cases of LG synesthesia in ten earlier studies (Bankieris & Simner, 2014; Colizoli, Murre, & Rouw, 2013; Gendle, 2007; Jones et al., 2011; Ramachandra, 2016; Richer et al., 2011; Simner & Haywood, 2009; Simner & Logie, 2007; Ward & Simner, 2003; Ward et al., 2005). All used the same validation method, known as a “test of consistency.” In this test, researchers presents LG synesthetes with a list of words (e.g., 80 words in Simner & Haywood, 2009) and require them to verbally describe their synesthetic flavor for each word (e.g., “table” = minced beef). A group of controls without synesthesia are required to assign a food to each word by free association. These word–food pairings are stored by the researcher, and the test is administered again to the same participants some time later (e.g., after 10 months have passed; Simner & Haywood, 2009). The researcher compares the flavors given during the test and retest, to determine whether the food association for each word was consistent over time (e.g., “table” = minced beef at both test and retest). Synesthetes are highly consistent (e.g., 70%–100% consistent across the word list), despite very long retesting intervals (typically several months, but even up to 30 years in one study: Simner & Logie, 2007). Controls are typically tested after a much shorter time interval (e.g., 2 weeks; Simner & Haywood, 2009) but still perform significantly worse than synesthetes. Indeed, controls perform poorly even if they are forewarned about the retesting or given financial incentives to do well (Ward et al., 2005). In our study, we took the spirit of this well-validated approach but innovated two novel versions, to addresses existing shortfalls.

There are several problems with the existing approach to testing. One is the time period between test and retest (e.g., 6 months), which makes diagnosis slow and effortful. Recent advances in other forms of synesthesia testing have shown that differences between synesthetes and nonsynesthetes can be detected even when the test and retest are given within a single session (e.g., Eagleman, Kagan, Nelson, Sagaram, & Sarma, 2007). This has worked well for synesthesia linking letters to colors; for example, a synesthete would see each letter three times within 15 min and select a color for each letter from an extensive digital color palette (e.g., with 16 million colors). This effective approach for color has never been applied to flavor, perhaps because verbally naming foods is quite different from selecting colors, and this raises concerns that controls might perform at ceiling from memory alone if they were retested for flavors within a single session. To address this concern, our diagnostic test here exploits single-session testing, while ensuring that our task is difficult enough to distinguish synesthetes from controls. A second problem for previous LG diagnostic tests is that they have been difficult to share widely, given differences from lab to lab in experimental software and testing interfaces. Our own test is run online and can be accessed from anywhere in the world that has an internet connection. Not only can researchers run the study in their own labs, but they can send the testing URL to participants so they can take part in their own homes.

A third problem in conventional LG testing is that it requires subjective interpretation: Researchers must judge whether two verbal utterances describe the same or different foods. The problem here is that LG synesthesia produces complex flavor sensations, meaning that the verbal description might change even if the flavor has not. J.I.W., for example, described one flavor as “meat fat” on one occasion but “bones and meat” on another. Another flavor was consistently breakfast cereal, but the brand had changed between test and retest. Should these be considered consistent? All this requires subjective judgments that not only introduce the possibility of error but require the time-consuming intervention of human coders. A fourth problem is that no studies have used an independently validated word list as the inducing stimuli. Importantly, some words are more likely than other words to trigger flavors. This means that any testing word list might be considered unsuitable if it happens to sample words that do not, on the whole, induce synesthesia flavors or suggest obvious flavors to nonsynesthetes. Our previous study (Ward & Simner, 2003) have shown that the presence or absence of synesthetic flavor is related to the linguistic features of the stimulus word: words that are common in the English language (cf. “pen” vs. “pun”) or words acquired before the age of 7 years (cf. “fairy” vs. “query”) are more likely to trigger flavors than words that are less common or are learned later. We used this information in our test design to ensure the best possible set of triggering words for our stimulus lists: All words were high in frequency (and familiarity) and were typically learned before 7 years. By this careful choice of stimuli, we could ensure that as many words as possible would stimulate synesthetic flavors in genuine synesthetes, making the test a more effective measure for the diagnosis of LG synesthesia.

In summary, we present a novel validated approach to the diagnosis of LG synaesthesia: a test that runs via an online interface, uses a carefully selected pool of stimulus words, evaluates consistency without human intervention, and makes a diagnosis within a single test session. We present two versions of our test here, which we pitted against each other to find the most effective diagnostic for LG synesthesia—not only in group-wise comparisons, but in whether the test allows an effective threshold score to separate synesthetes from nonsynesthetes (see below). In each test, we presented a 30-item word list and required synesthetes to describe their food association for each word. These 30 words were presented again in an immediate retest within the same testing session, and the consistency of the food responses was compared word by word in an automated way across test and retest. In Experiment 1, participants described their synesthetic flavors by selecting the related food name from a comprehensive hierarchical display (e.g., Is it a meat? If so, is it chicken? beef? pork? etc.). In Experiment 2, participants described their food association according to its five basic tastes (i.e., How salty is it? How sweet? How bitter? How sour? How umami?).

We applied receiver operating characteristic (ROC) analyses to our data to examine how effective each test was at successfully detecting synesthetes (i.e., the test’s “sensitivity”) and successfully rejecting nonsynesthetes (i.e., its “specificity”). To anticipate our results, we found that both methods produced significant group differences in the consistency scores of those who did versus those who did not self-report synesthesia, although our second test (Exp. 2) had greater diagnostic value in better differentiating synesthetes from nonsynesthetes with a threshold cutoff.

## Experiment 1: Diagnosing LG synesthesia using food categories

### Method

#### Participants

Our 85 participants comprised 28 self-declared LG synesthetes (26 females, two males, mean age = 46.21 years, *SD* = 14.43) and 57 self-declared nonsynesthetes (40 females, 17 males, mean age = 48.32 years, *SD* = 16.39). An independent-samples *t* test showed no significant differences between the groups in age [*t*(83) = 0.577, *p* = .566]. Our synesthetes were recruited from our database of synesthete participants who had previously contacted the University of Sussex to offer to take part in our synesthesia research, and via the UK Synesthesia Association, whom they had previously contacted to report their LG synesthesia. The control participants were recruited through advertisements in the media and from Prolific.ac, an online participant recruitment platform that holds a database of individuals who have expressed an interest in taking part in research studies. Both experiments presented here were approved by the local university ethics committee, and the study was conducted in accordance with the ethical standards laid down in the 1964 Declaration of Helsinki.

#### Materials

Word stimuli were 30 words in English (mean length = 6, *SD* = 1.86, range = 3–10), typically acquired between the ages of 3 and 6 years (mean age-of-acquisition [AoA] rating = 301.30, *SD* = 52.14, range = 206–381). The words were especially common words in English, with an average CELEX word frequency of 115.23 (*SD* = 48.82, range = 57.65–248.88; Baayen, Piepenbrock, & Gulikers, 1995) and a mean familiarity rating of 579.63 (*SD* = 26.95, range = 500–630; Davis, 2005; Gilhooly & Logie, 1980; Toglia & Battig, 1978).

**Fig. 1:**
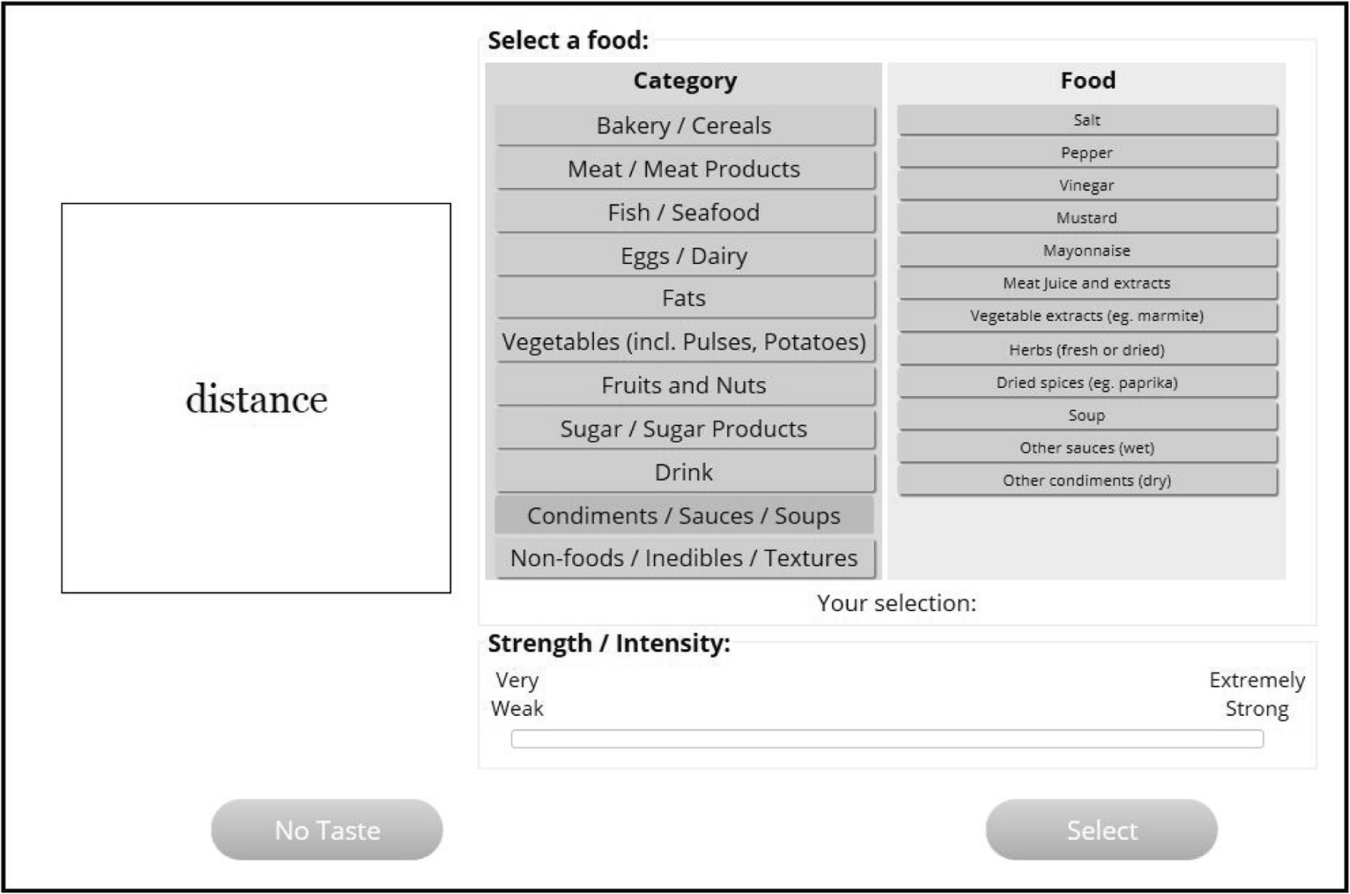
Testing interface for the objective consistency test in Experiment 1. The example is based on the target word “distance” and a response made from the “Condiments/Sauces/Soup” category

Participants also saw a palette of food names, divided hierarchically into superordinate and subordinate categories. This food palette was based on the DAFNE (Data Food Networking) Food Classification System, used in the UK and throughout Europe (http://ec.europa.eu/health/ph_projects/2002/monitoring /dafne_code_en.pdf). Minor changes were made to reflect the food experiences that are often described by LG synesthetes (see Ward & Simner, 2003). For example, synesthetes’ flavors are weighted toward sugary produce and chocolate, so the category of “Sugar/Sugar products” was expanded in this regard. Table 1 shows the final palette of foods, and Fig. 1 shows an example of the way these foods were hierarchically presented on screen during our test. Before running the study, we ran a pilot study that tested the usability of the test interface, to ensure that individuals would be able to consistently report tastes using it. The data from this pilot study can be found in the supplementary materials.

**Table 1:** Foods (superordinate food categories) used as Experiment 1’s food palette

Bakery/Cereals Bread and rolls Other bakery products VFlour Pastry Pasta Breakfast cereals Rice/other cereals (excl. sweet corn)	Meat/Meat Products Pork/bacon Beef/veal Other red meat Offal Poultry Meat products (e.g., sausage; canned) Other meat dishes	Fish/Seafood Fish, fresh/frozen/processed (e.g., tinned) Seafood Fish dishes (e.g., breaded fish)
Eggs/Dairy Eggs Milk Cheese Other milk products (e.g., Yoghurt)	Fats Butter Other animal fat Vegetable fat (e.g., margarine) Vegetable oil (e.g., olive, sesame)	Sugar/Sugar Products Sugar Chocolate Sweets/ Candy Artificial sweetener Other sugar products
Vegetables (incl. Pulses, Potatoes) Cabbage Other green leafy Cucumber Tomatoes Carrots Mushrooms Peppers/chilis Squash (e.g., pumpkin) Broccoli Onions/garlic/leek Potatoes/other starchy root Beans (e.g., green, baked) Other pulses (e.g., peas, lentils) Other vegetables (incl. sweet corn)	Fruits and Nuts Apples Citrus Bananas Grapes Plums Berries (e.g., strawberry) Apricots/peaches Cherries Pears Nuts or peanuts Dried/processed fruits Other fresh fruits	Condiments/Sauces/Soups Salt Pepper Vinegar Mustard Mayonnaise Meat Juice and extracts Vegetable extracts (e.g., marmite) Herbs (fresh or dried) Dried spices (e.g., paprika) Soup Other sauces (wet) Other condiments (dry)
Beverages Coffee Tea Cocoa Water Fruit/ vegetable juice Other soft drink (excl. milk) Wine Beer Spirit	Nonfoods/Inedibles/Textures Medication' Organic (e.g., earwax) Inorganic/chemical (e.g., plastics) Texture: Rough/hard/crunchy Texture: Smooth/soft/chewy Temperature: Warm/hot Temperature: cold Shape: Nonfood Other (e.g., an action) Distinct but cannot identify

#### Procedure

Participants were tested remotely via an online interface hosted on our testing platform, *The Synaesthesia Toolkit*, and entered the test by clicking on its URL. On entering the test, participants first provided demographic information, such as age and gender. Participants then proceeded to the main test, which screened for synesthesia in the two-step process of a self-report questionnaire followed by an objective test of consistency.

##### Self-report questionnaire

Participants read the following description about synesthesia, and were then required to self-report whether or not they experienced LG synesthesia:

This study is looking at synaesthesia, a rare condition that causes a kind of “merging of the senses.” We are interested in **taste**^1^ synaesthesia, a condition where thinking about words causes unusual taste sensations. For example, hearing the word “door” might trigger the taste of blackcurrants. Synaesthesia is rare and not many people have it. Synaesthesia is NOT the kind of associations everyone makes. E.g. the word “tin” or “can” probably make everyone think of beans or peas or coke. This is NOT synaesthesia. Synaesthesia is automatically linking words to foods, even if the word isn’t normally related to food at all. In synaesthesia, tastes can flood the mouth (like real tastes), or even just be strong thoughts that come automatically to mind. For example, hearing the word “door” might trigger the taste of blackcurrants in the mouth, or the thought of blackcurrants in the mind. Both are synaesthesia (so long as it’s automatic and has happened a lot since childhood).

**Fig. 2:**
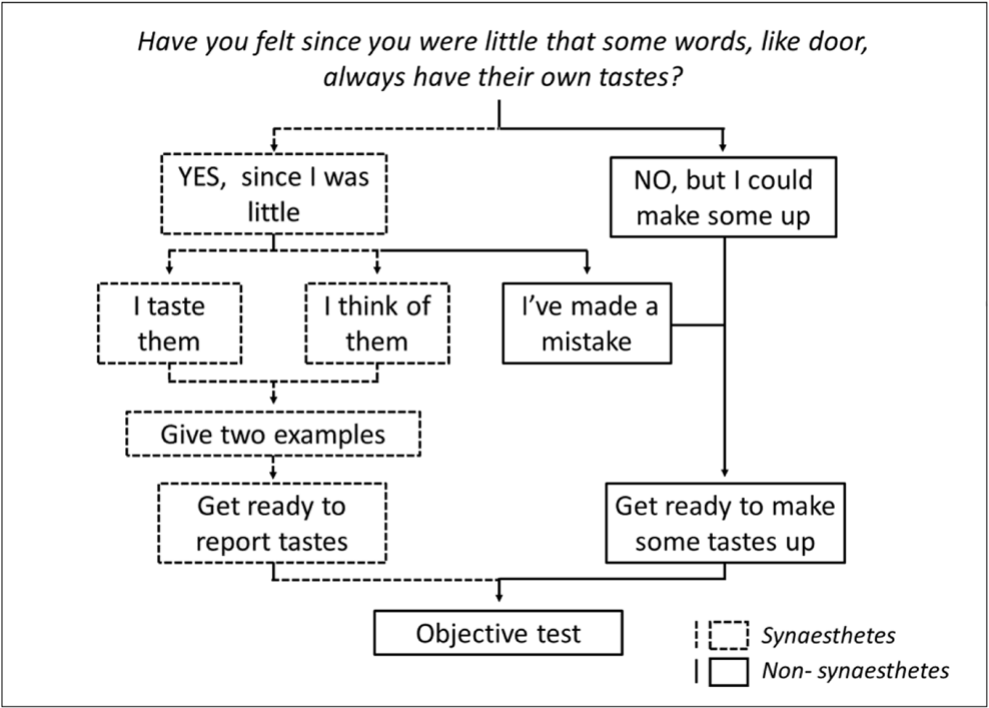
Diagram of synesthete (dashed lines and gray fill) and nonsynesthete (solid lines and no fill) responses to the self-report questions

Participants were then asked the following question, to allow them to self-report having or not having synesthesia: *“Have you felt since you were little that some words, like ‘door,’ always have their own tastes? (even if the words aren’t related to food at all).”* They responded by ticking either: *“YES, I’ve thought this since I was little”* or *“NO, not really . . . but I could probably make some up today if I tried.”* If participants answered “no,” they were told they would be required to invent word–food associations. If they answered “yes,” they were prompted to indicate whether they experienced the food association as a veridical flavor in the mouth (which we refer to in our analyses as *projector* synesthesia) or as thoughts in the mind (referred to as *associator* synesthesia). A third option was the chance for the participant to reject his or her previous self-report of synesthesia (i.e., *“I’ve made a mistake – I DON’T feel that words have their own tastes”*). If one of the first two options was chosen (i.e., “flavors in the mouth” or “thoughts in the mind”), participants were asked to provide two examples of a word and the flavor it triggered. If participants stated they had made a mistake, they were shown the same text presented to those who answered “no” to having synesthesia. Following this, all participants clicked to begin the objective consistency test. Figure 2 outlines the flow of the questions and the possible responses for synesthetes and nonsynesthetes.

##### Objective consistency test

Participants were given the following instructions: “In this test, we will show you a list of words and ask you to think of a taste for each word. The taste can be a food or drink etc. E.g. if we give you the word ‘filter,’ you might associate this with the taste of coffee.” The individuals classed as nonsynesthetes on the basis of their questionnaire response were given the additional instructions to just invent these associations (“Just read the word and think of the first taste that comes to mind. We know this is an unusual thing to ask but we want you to get creative!”). Words were presented onscreen individually alongside our food palette. Participants were required to select their food association from the palette by first clicking on a food category and then selecting one of the subordinate foods within that category. Figure 1 shows a screenshot based on the example target word “distance” and the interface seen as if a participant selected the food category “Condiments/Sauces/Soups.”

Participants were also asked to rate the strength/intensity of the association, on a scale from *Very weak* to *Extremely strong*, using a slider. There was no preset value, and a response marker appeared on the scale only when participants had clicked on it. Participants were told they could press a “no-taste” button if it was impossible for them to answer, but they were urged not to press the button too often and to try hard to think of a flavor for each word, even if the flavor association was not instantly obvious. Participants clicked “Select” when they were ready to move on to the next trial, in which case the screen would not advance until they had selected a subordinate food (e.g., mayonnaise) and an intensity rating, or they selected “No taste.” Participants completed two blocked repetitions of the word list. Words were fully randomized within each block. Once the participant had responded to each of the 30 words twice, they were debriefed and thanked for their participation.

### Results

#### Self-report questionnaire

As expected, all the LG synesthetes, and no controls, self-reported having LG synesthesia. Within the LG synesthetes, 11 reported having associator synesthesia, and 17 reported having projector synesthesia.

#### Objective consistency test

Our two aims were to determine whether our test of consistency would (a) discriminate group-wise between self-declared LG synesthetes and nonsynesthetes, and (b) provide a useful threshold cutoff for future test users, to effectively diagnose LG synesthesia in new individuals.

#### Scoring the test

For each participant, we compared food responses to the first and second presentations of each word (e.g., we compared the responses for the first and second presentations of the word “distance”). A score of 2 points was awarded for an exact match across the two presentations (i.e., the same category and the same subordinate food; e.g., “Fats/Butter”–“Fats/Butter”). A score of 1 was awarded for a partial match [i.e., same food category but different subordinate foods; e.g., “Fats/Butter”–“Fats/Vegetable fat (e.g., margarine)”]. The total number of consistent trials excluding “no-taste” responses was converted to a percentage, out of the maximum number of available points. For example, a participant responding with four consistent foods, one partial match, five inconsistent foods, and 20 no-taste responses would score nine points out of a possible 20 (2 points available for each of the ten words for which at least one food was provided) and would be given a score of 45.00%. We excluded consistent no-taste responses in order to prevent highly consistent datasets that would consist predominantly of no-taste responses (e.g., in the previous example, this poor-performing participant would otherwise have scored 81.70%, because they would have scored a further 40 points from consistent “no-taste” responses, and the total of 49 points would be scored out of 60, the sum of 2 points per every trial). The intensity responses were scored from 1 (*Very weak*) to 100 (*Extremely strong*), with 0 being assigned to any word that was given a no-taste response on one presentation and a taste response on the other.

#### Analyses

**Fig. 3:**
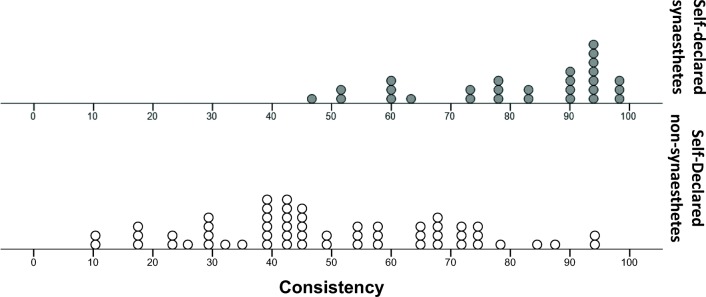
Distribution of consistency scores from the food-category task, for self-declared synesthetes (top) and nonsynesthetes (bottom). Each point represents one participant’s score. Participants were awarded 1 point for partial matches and 2 points for exact matches. These points were summed and divided by the total available score (number of words that were given a flavor in at least one presentation, multiplied by 2)

Figure 3 shows the distributions of consistency scores for our two groups of participants. We compared the groups using nonparametric tests because the scores were nonnormally distributed for synesthetes, *W*(28) = 0.88, *p* = .005. We found that the LG synesthetes were significantly more consistent (Mdn = 85.90%) at reporting flavor associations than were the nonsynesthete controls (Mdn = 45.00%), *U* = 203.00, *p* < .0005, *r* = .60. However, despite the group difference, Fig. 3 shows that no clear cutoff value separates synesthetes from nonsynesthetes.

To rule out the possibility that the number of words to which participants assigned tastes might have accounted for the difference in performance across the synesthete and nonsynesthete groups, we ran a two-step hierarchical linear regression, predicting consistency scores from the percentage of words given tastes on both list presentations and from synesthete status. The first model was significant, *F*(1, 83) = 4.78, *p* = .032, explaining 5.00% of the variability in consistency scores; as the number of words with assigned tastes, *β* = – .23, *t* = – 219, *p* = .032, decreased, consistency increased. The addition of synesthete status as a predictor resulted in another significant model, *F*(2, 83) = 23.30, *p* < .0005, this time explaining 36.20% of the variability in consistency scores. The change in the percentage of variability explained was significant (*p* < .0005). Crucially, once synesthete status was added to the model, it became the only significant predictor in the model, *β* = .59, *t*(82) = 6.29, *p* < .0005, and the percentage of words given tastes no longer significantly predicted the consistency score, *β* = – .03, *t* = – 0.31, *p* = .759. Overall, this shows that the group-difference in the number of words with tastes did not account for the relationship between consistency and synesthete status, because although synesthetes assigned tastes to significantly fewer words, and although the number of “tasty” words predicts consistency score, synesthete status explained significantly more variability in consistency scores than the number of words with tastes did.

**Fig. 4:**
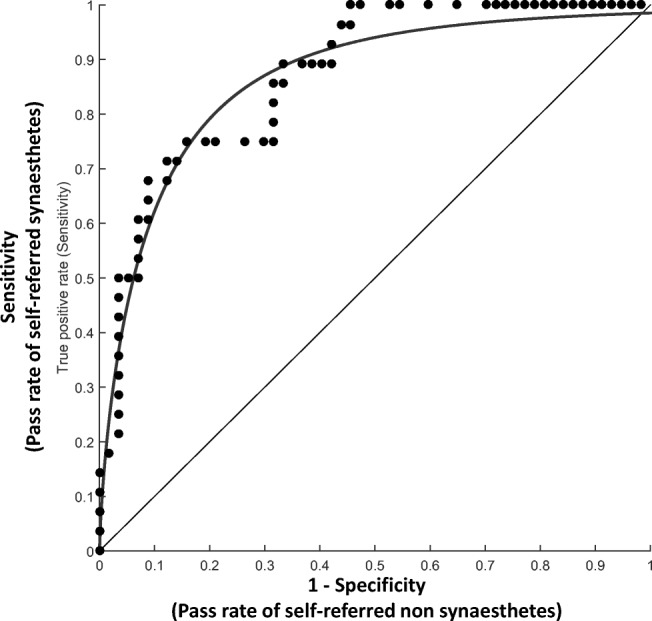
Receiver operating characteristic (ROC) curve showing the trade-off between sensitivity and specificity of the categories task in predicting self-declared synesthesia, at different cutoff values (curved line). The straight diagonal line represents a test with no discriminant power (i.e., that classifies scores at a guessing rate), for comparison. The dots represent sensitivity (*y*-axis) and 1-specificity (*x*-axis) values for each *R*-square score. *Sensitivity* represents the probability of detecting synesthesia in self-declared synesthetes, whereas *1-specificity* is the probability of incorrectly passing self-declared nonsynesthetes. The optimal cutoff value is defined as the point that results in the highest hit rate (i.e., is highest on the vertical axis) and the lowest false alarm rate (horizontal axis). The area under the curve represents the discriminant power of the test

To explore this result further, we applied receiver operating characteristics (ROC) analysis to the data, to examine how effective our test is at predicting participants’ status as an LG synesthete or nonsynesthete. We used self-reports to classify the presence and absence of synesthesia and used consistency scores as a predictor. The analysis computed a continuum of potential cutoff scores (see Fig. 4) that can be used for a diagnostic test, and for each one provided measures of sensitivity and specificity. *Sensitivity* is represented by the proportion of self-declared synesthetes with consistency scores greater than the cutoff (i.e., hits), and *1-specificity* is represented by the proportion of nonsynesthetes with consistency scores greater than the cutoff (i.e., false alarms). The area under the curve (AUC) is taken to represent the overall predictive accuracy of a diagnostic tool. This statistic runs linearly from .5 (guessing rate) to 1 (perfect predictive power). Our consistency test yielded an AUC of .86, *p* < .0005, *SE* = .05, 95% CI [.77, .96], indicating good but not excellent predictive power.

Our analysis revealed that maximum sensitivity (i.e., classifying all self-declared synesthetes as synesthetes) would come with a score threshold of 45.83% (see Table 2 for the sensitivity and specificity values corresponding to each cutoff score value). This threshold would, however, also classify 45.61% of self-declared nonsynesthetes as synesthetes. A threshold of 95% would achieve maximum specificity (i.e., it would classify all those individuals who reported not having synesthesia as nonsynesthetes), but it would also classify 85.71% of self-declared synesthetes as nonsynesthetes. On the basis of our data, the cutoff with maximum efficiency—that is, the test threshold score that would pass the largest number of self-declared synesthetes (67.86%) while also passing the smallest number of nonsynesthetes (8.77%)—is 75%.

**Table 2:**
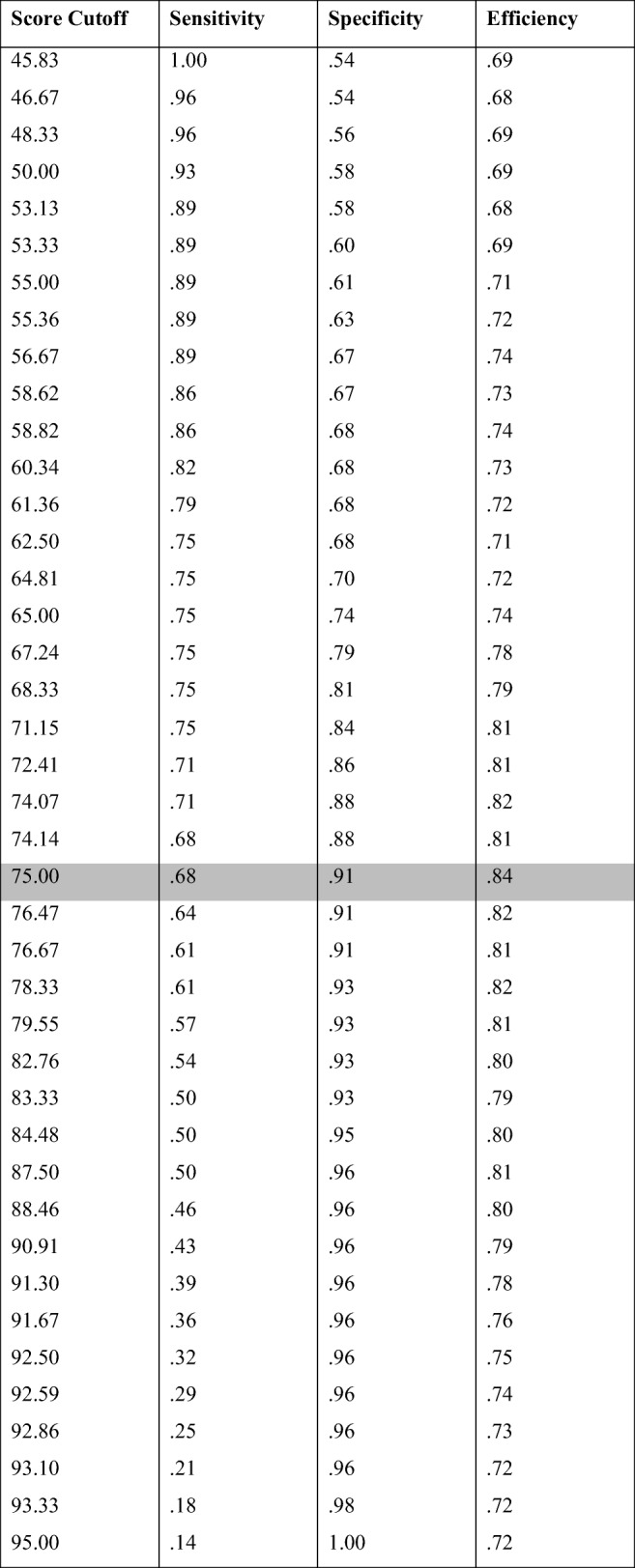
Sensitivity and specificity values for increasing category cutoff scores, ranging from sensitivity = 1 to specificity = 1. The cutoff (75.00%) with the maximum efficiency is highlighted in gray. Sensitivity represents the probability of detecting synesthesia in self-declared synesthetes, whereas specificity is the probability of correctly rejecting self-declared nonsynesthetes. Efficiency represents the proportion of cases classified in line with self-report

We also looked at whether the consistency of food choices separated projector from associator LG synesthetes. The data were not normally distributed for either associators, *W*(11) = .82, *p* = .019, or projectors, *W*(17) = .88, *p* = .029, so a nonparametric test was used. There was no significant difference between associators (Mdn = 91.66) and projectors (Mdn = 83.33) in this measure of consistency, *U* = 79.00, *p* = .517, *r* = .13.

**Fig. 5:**
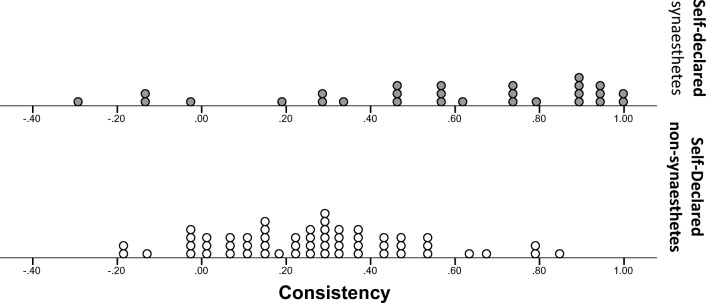
Distribution of our consistency dependent measure for intensity ratings given across the two presentations of the word list. For each individual, scores were computed by correlating the intensity ratings given for each word across the two presentations

We next examined participants’ consistency at rating the intensity of flavor associations across the two presentations of the word list. To calculate our dependent measure for the consistency of intensity, we correlated the intensity ratings given by each participant in the first presentation with those given in the second presentation, for the same words. Hence, our intensity consistency measure (a correlation coefficient) ranged from – 1 to 1. When a no-taste response was given on only one of the two presentations, an intensity of 0 was assigned to the word and was correlated against the intensity given for the taste response in the other presentation. If no-taste responses were given in both presentations of the same word, the trial was not included in the correlation. This was again done to avoid data sets with a small number of inconsistent responses attaining a high score due to the predominance of no-taste responses. The distribution of these scores as a function of self-declared synesthete status can be seen in Fig. 5. The synesthete data were not normally distributed, *W*(28) = .910, *p* = .019, and variance was heterogeneous across groups, *F*(1, 83) = 8.84, *p* = .036, so nonparametric comparisons were used. On average, the measures of the correlation between intensity ratings given on the first and second presentations of the word list were significantly higher in the synesthete group (Mdn = .60) than in the nonsynesthete group (Mdn = .27), *U* = 390.00, *p* < .0005, *r* = .41. However, a ROC analysis of the intensity correlation scores and self-declared synesthete status showed that intensity scores did not fare any better at discriminating between self-declared synesthetes and nonsynesthetes than did our previous measure: AUC = .76, *p* < .0005, *SE* = .06, 95% CI [.64, .87]. Finally, we note that there were no differences in the consistency of intensity across associators (*M* = .55, *SD* = .41) and projectors (*M* = .56, *SD* = .36), *t*(26) = 0.073, *p* = .942, Cohen’s *d* = 0.03.

Above we saw that LG synesthetes were more consistent in their intensity ratings, but they also gave higher ratings overall: we looked at the average intensity ratings (on a scale from 0 to 100) within each presentation of the word list, and ran a mixed 2×2 analysis of variance crossing word list presentation (first, second) and group (synesthete, nonsynesthete). Although there was no significant effect of presentation, *F*(1, 83) = 1.12, *p* = .292, *η*_p_^2^ = .01, and no significant interaction, *F*(1, 83) = 0.78 *p* = .375, *η*_p_^2^ = .01, we did observe a main effect of group, *F*(1, 83) = 14.93 *p* < .0005, *η*_p_^2^ = .15. This indicated that flavor associations were significantly stronger for self-declared synesthetes (*M* = 57.43, *SD* = 19.70) than for nonsynesthetes (*M* = 39.86, *SD* = 19.70). Within our group of LG synesthetes, associators (*M* = 60.03, *SD* = 12.13) and projects (*M* = 55.75, *SD* = 14.66) reported similar levels of intensity; we found no group difference in the intensity of word–taste associations, *F*(1, 26) = 0.37, *p* = .548, *η*_p_^2^ = .01, no main effect of presentation, *F*(1, 26) = 0.02, *p* = .887, *η*_p_^2^ = .001, and no interaction, *F*(1, 26) = 0.02, *p* = .880, *η*_p_^2^ = .001.

### Discussion

In our experiment, we tested a group of self-declared LG synesthetes and self-declared nonsynesthetes. Our test aimed to distinguish synesthetes from nonsynesthetes using a consistency measure in which words are associated with foods selected from a hierarchical list of food names. Words were presented twice, and we calculated the consistency with which the same words were given the same food association for each participant. We found that the synesthete group was significantly more consistent in their food associations across test and retest, and they were also significantly more consistent when ratings the intensity of those word–food associations. Synesthetes also rated their flavors as being more intense overall. Finally, when we looked within our group of LG synesthetes, we found that associators and projectors performed similarly on every measure.

We might also conclude that we selected our target words well. Firstly, the synesthetes provided synesthetic tastes for 83% of the words in Experiment 1, and for 87% in Experiment 2. These hit rates are high in comparison to the low rates previously recorded from LG synesthetes in other studies (e.g., less than 60% in the word list of Ward et al., 2005). Secondly, all 30 words elicited a taste from at least 50% of synesthetes in Experiment 1, and from at least 38% in Experiment 2, with the majority of words (27/30) eliciting a taste response in more than half of the synesthete sample.

Although our test showed a number of group-wise differences, there was some degree of overlap in the consistency with which food associations were given over time, across synesthetes and nonsynesthetes. Our ROC analysis showed good, but not excellent, discriminability. A threshold high enough to recognize at least eight out of ten self-declared synesthetes (a score of approximately 60%) would nonetheless have a 32% chance of classifying nonsynesthetes as synesthetes. Reducing this error rate to only 8% would only pass around 6.7 out of ten of the self-declared synesthetes. For this reason, we present an alternative way to diagnose LG synesthetes below.

## Experiment 2: 5-Tastes pie chart

**Fig. 6:**
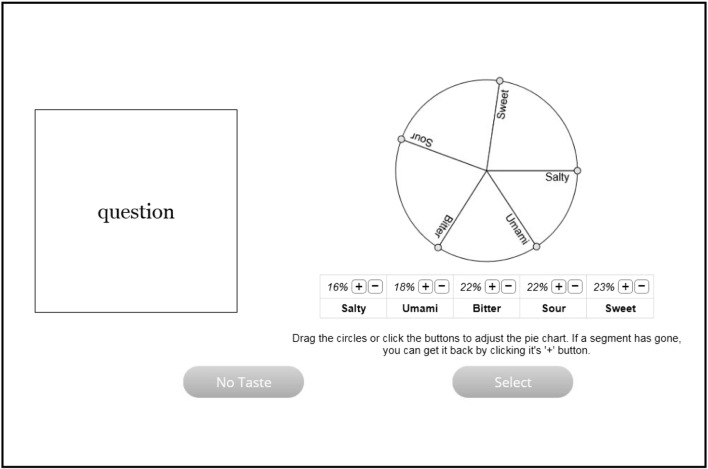
A single trial in Experiment 2, showing the target word on the left (here, “question”) and the taste selection pie chart on the right

In Experiment 2, we again introduce an online test for LG synesthesia, but each food is now selected by describing it in terms of its five basic tastes (sweet, salty, bitter, sour, and umami). After deciding on their food association, participants now adjusted five segments of a pie chart, one for each taste, to show the relative contributions of each taste to the overall flavor of the food (see Fig. 6, in methods section). For example, if a participant associated the word “America” with the flavor of a cheeseburger, they would ask themselves how the flavor of a cheeseburger breaks down into the five basic tastes. For example, they might rate it as being mostly umami (i.e., meaty), then salty, a bit sweet, and a bit sour from the relish. The taste would not be bitter at all (unless the burger was burnt). The participant could then adjust the taste pie chart accordingly, making umami the largest segment, then salty, and so on.

We point out that our pie-chart method measures the relative contribution of each of the five basic tastes, but it would equally have been possible to elicit absolute ratings for the five basic tastes separately, in five independent Likert scales. These would produce very different scores. Consider, for example, that the confectionary “lemon drops” might be rated on five independent Likert scales as 80% sweet and 80% sour and 0% umami, salty, and bitter; this would indicate that it was very sweet and very sour. But within a pie chart, the values must sum to 100%, meaning that it would likely be rated 50% sweet and 50% sour (again with 0% umami, salty, and bitter). Hence, the pie chart does not tell us the absolute sweetness or sourness, but rather that these two tastes contribute equally to the overall flavor. Our choice of a pie chart over Likert scales was made carefully, given our recent study (Hughes et al., in prep) that had shown that controls struggled disproportionately more when making this type of relative cross-modal judgment than did synesthetes.

In summary, we present below a second way to assess LG synesthesia, again using an online interface and self-report questionnaire, but with a new method for indicating foods in the objective consistency test. As before, we measured how effective our interface was in distinguishing synesthetes from controls.

### Method

#### Participants

Our 64 participants comprised 21 self-declared synesthetes (19 females, two males, mean age = 47.95 years, *SD* = 14.04; ten associators and 11 projectors) and 43 self-declared nonsynesthetes (35 females, eight males, mean age = 48.84 years, *SD* = 16.62). Participant groups were matched on age, *t*(62) = 0.210, *p* = .834, Cohen’s *d* = 0.06. Thirty-eight of these participants (18 synesthetes and 20 nonsynesthetes) also took part in Experiment 1, and all new participants were recruited from the same populations as before. For those who took part in both tests, the order of testing was counterbalanced across participants.

#### Materials

Stimuli were a new list of 30 words in English, to ensure that they were equally unfamiliar to all participants (mean length = 5.5, *SD* = 1.78, range = 3–10). As in Experiment 1, these words were high in frequency and familiarity, and were typically learned at a young age. The average CELEX word frequency was 202.51 (*SD* = 128.32, range = 54.86–496.87), and the mean familiarity rating was 580.57 (*SD* = 33.64, range = 473–627). AoA measures showed that these words are typically acquired between the ages of 3 and 7 years (mean AoA rating = 306.50, *SD* = 61.49, range = 222–447). Participants responded to these words using a carefully designed online interface. This was a manipulable pie chart divided into five segments labeled *Sweet*, *Sour*, *Salty*, *Bitter*, and *Umami*. This pie chart is described in detail in the Procedure section. As in Experiment 1, we first ran a pilot study that tested the usability of the test interface, to ensure that individuals would be able to consistently report tastes using it. The data from this pilot study can be found in the supplementary materials.

#### Procedure

Participants were again tested using an online interface that first gathered demographic information and then presented the same preamble and examples describing LG synesthesia from Experiment 1. As before, we next presented a screening test for synesthesia, using the same two-step structure of a self-report questionnaire followed by an objective test of consistency. The self-report was identical to that in Experiment 1, but the objective test of consistency was different. It again presented 30 words individually onscreen and required participants to associate a food to each word. And it again presented two (blocked) repetitions of the word list, fully randomized within each block as before. However, participants now indicated their food association in a different way, by describing its five basic tastes. Participants were told to indicate the relative taste qualities of the food on a pie chart divided into five slices, each labeled “sweet,” “sour,” “bitter,” “salty,” and “umami,” respectively. On each trial, participants saw the target word on the left of the screen and the pie chart on the right (see Fig. 6).

The starting values on the pie chart at the beginning of each trial were chosen randomly and were always assigned values equal to or greater than 1. Participants were instructed to adjust the pie chart by dragging the segment dividers until the pie chart reflected the flavor of the food they had thought of. Beneath the pie chart, the five labels were repeated horizontally, along with the percentage that reflected the size of the slice for each label on the pie chart. Above each label (e.g., above “Sweet”) were a plus (“+”) and a minus (“–”) button, which offered a second way to adjust the pie chart. Pressing the “+” button would increase the percentage of the pie chart taken up by that particular taste, whereas “–” would decrease the percentage (and the pie-chart segments would change in size accordingly). Once participants had seen all the words twice, the test displayed a debrief describing the purpose of our study. There was no time limit to complete the task.

Prior to starting the test, instructions were given very carefully, with clear examples. The instructions included the following text:

In this test, we will show you a list of words and ask you to think of a taste for each word. The taste can be a food or drink etc. E.g. if we give you the word “America,” you might associate this with the taste of cheeseburger. But we would like you to describe that food with the 5 basic tastes of:

Sweet, Salty, Umami, Sour, Bitter

(Please click on each taste to read its definition).

So we want you to tell us your food/drink association for each word we give you, by describing the food in its basic tastes. For example if we give you the word “America” you might associate this word with the taste of a cheeseburger. So what is the taste of a cheeseburger? It is mostly umami (i.e. meaty), then salty, a bit sweet and a little bit sour from the relish. The taste won’t be bitter at all (unless your burger is burnt!)

During this instruction phase, participants were invited to click on each of the five basic tastes to reveal a popup window showing definitions and examples of these five tastes, if they were unsure. These definitions (see Table 3) also included an explicit explanation of the difference between bitter and sour, given the propensity for participants to confuse these two tastes (Meiselman & Dzendolet, 1967; O’Mahony, Goldenberg, Stedmon, & Alford, 1979). Our definitions and examples are shown in Table 3.

**Table 3:**
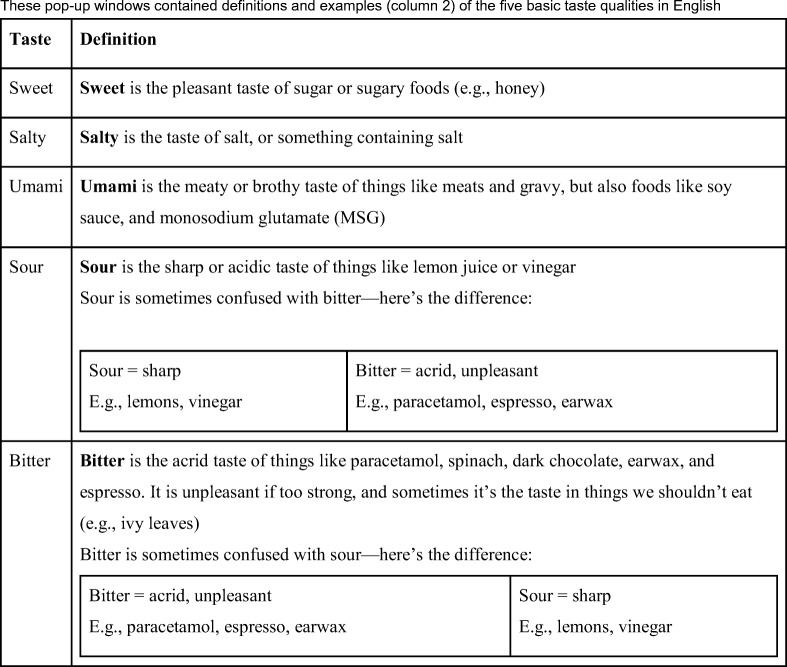
Labels of the five on-screen buttons (column 1) that revealed popup windows during our instructions

### Results

Our aims were again to determine whether our test of consistency would discriminate group-wise between self-declared LG synesthetes and nonsynesthetes, and whether it could provide a useful threshold cutoff (appropriate in sensitivity and specificity) for future test users to effectively diagnose LG synesthesia in new groups.

#### Scoring the test

Consistency across repetitions was calculated by regressing responses collected during the first presentation of words against the responses from the second presentation for the same words, for each of the five tastes. The five resulting *R*^2^ values were then averaged to produce one mean value, and then converted into a percentage. This percentage score represented the average percentage of variability in the second set of responses explained by the variability in the first set. In other words, the consistency score reflects how well the first set of responses predicted the second set. In cases in which a taste response was given on one presentation but a no-taste response on the other presentation of the same word, the no-taste response was replaced with values of 0 for all five tastes. Because running a regression on a small number of cases can yield unreliable *R*^2^ estimates, taste categories that were not assigned tastes on more than 10% of words (i.e., > 3 words) were not included in the average consistency score, and the score was calculated using the remaining categories. For example, if “bitter” was given 0% on all but one or two words, the consistency score would be the average of the *R*^2^ values from the “sweet,” “sour,” “salty,” and “umami” responses. This occurred in two control data sets and 12 synesthetes. We point out that our step here did not make, on average, a significant difference to the scores of those data sets affected (before step, *M* = 68.14, *SD* = 30.00; after step, *M* = 69.14, *SD* = 26.92), *t*(14) = – 0.46, *p* = .652, Cohen’s *d* = 0.13; in other words, although this step affects the responses of more synesthetes than nonsynesthetes, it does not give synesthetes any advantage, because on average the scores were the same after exclusion of the taste categories.

#### Analyses

**Fig. 7:**
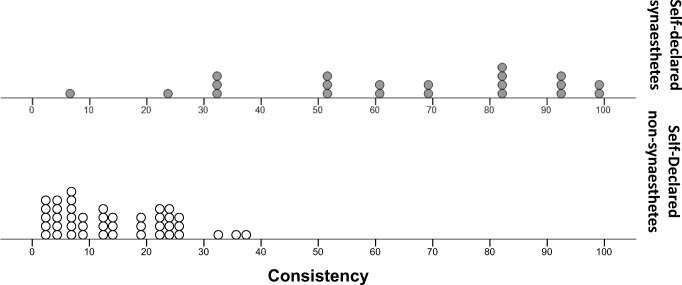
Distributions of *R*^2^ consistency scores for self-declared synesthetes (top) and nonsynesthetes (bottom). Scores were calculated by regressing the responses given on the first presentation against the responses from the second presentation of each taste, and then averaging across the five tastes and converting to a percentage. Each point on the distribution represents one score

We examined whether the two groups showed different levels of consistency when describing their taste associations. Our data were not normally distributed in the nonsynesthete group, *W*(43) = .93, *p* = .008, and the variance across groups was heterogeneous, *F*(1, 62) = 36.42, *p* < .0005, so the analyses were run using nonparametric tests. Synesthetes were consistently more consistent than nonsynesthetes. In other words, synesthetes’ responses to Presentation 1 were significantly more predictive of those in Presentation 2 (Mdn = 67.77) than was true among nonsynesthetes (Mdn = 13.25), *U* = 46.00, *p* < .0005, *r* = .72 (see Fig. 7 for the distribution of scores).

As in Experiment 1, we ran a two-step hierarchical linear regression, predicting consistency scores from the percentage of words that were assigned tastes and from synesthete status. The first model was significant, *F*(1, 62) = 22.30, *p* < .0005, with the percentage of words assigned tastes, *β* = – .51, *t* = – 4.72, *p* < .0005, explaining 26.5% of the variability in consistency scores. The addition of synesthete status as a predictor also resulted in a significant model, *F*(2, 61) = 59.05, *p* < .0005, that explained 65.90% of the variability in consistency scores. The change in variability explained was significant (*p* < .0005). Crucially, once synesthete status was added to the model, it became the only significant predictor in the model, *β* = .74, *t* = 8.41, *p* < .0005, and the percentage of words given tastes no longer significantly predicted consistency score, *β* = – .12, *t* = – 1.36, *p* = .177. Overall, this confirms that although synesthetes assigned tastes to significantly fewer words than did nonsynesthetes, and the number of words to which participants assigned tastes was related to consistency score, synesthete status explained significantly more variability in consistency scores than did the number of words with tastes, and thus the difference in the number of words with tastes across the two groups does not account entirely for the relationship between consistency and synesthete status.

**Fig. 8:**
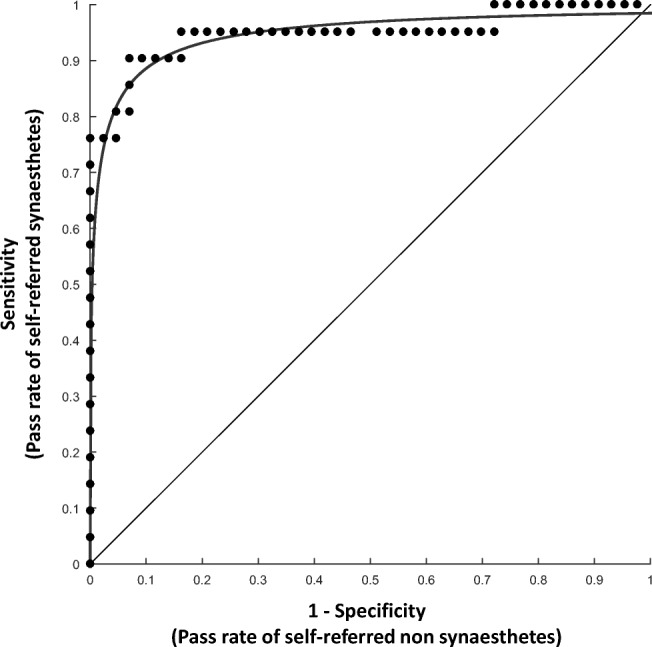
Receiver operating characteristic (ROC) curve showing the trade-off between sensitivity and specificity of the 5-Tastes task in predicting self-declared synesthesia, at different cutoff values (curved line). The straight diagonal line represents a test with no discriminant power (i.e., that classifies scores at guessing rate), for comparison. Dots represent sensitivity and one-specificity values for each *R*-square score. The optimal cutoff value is defined as the point that results in the highest true positive rate (i.e., is highest on the vertical axis) and the lowest false positive rate (horizontal axis). The area under the curve represents the discriminant power of the test

We next asked whether our test differentiated not only group-wise, but also with a useful threshold cutoff, in terms of its sensitivity and specificity. We applied ROC analysis to the data using self-reports to classify the presence and absence of synesthesia and using consistency scores as a predictor to examine how effective the consistency test was at predicting participants’ self-reports of synesthesia. The analysis showed that the test had excellent predictive power, AUC = .945, *p* < .0005, *SE* = .036, 95% CI [.88, 1.00]. Figure 8 shows the ROC curve, fitted to sensitivity plotted as a function of 1-specificity, for each score.

Our analysis revealed that maximum sensitivity (i.e., classifying all self-declared synesthetes as synesthetes) would come with a score threshold of 6.3% (i.e., the worst score of synesthetes was 6.3%). This threshold would, however, also classify 72.91% of the self-reported nonsynesthetes as synesthetes. On the other hand, a threshold of 37.40 would achieve maximum specificity (i.e., it would classify all those individuals who reported not having synesthesia as nonsynesthetes), but would also classify 23.81% of self-declared synesthetes as nonsynesthetes. With both these considerations, our test revealed that the maximum efficiency cutoff with a balance between these two extremes was 26.06%—that is, the test threshold score that would pass the largest number of self-declared synesthetes (90.48%) while also passing the smallest number of nonsynesthetes (6.98%; see Table 4 for the sensitivity and specificity values corresponding to each cutoff score value).

**Table 4:**
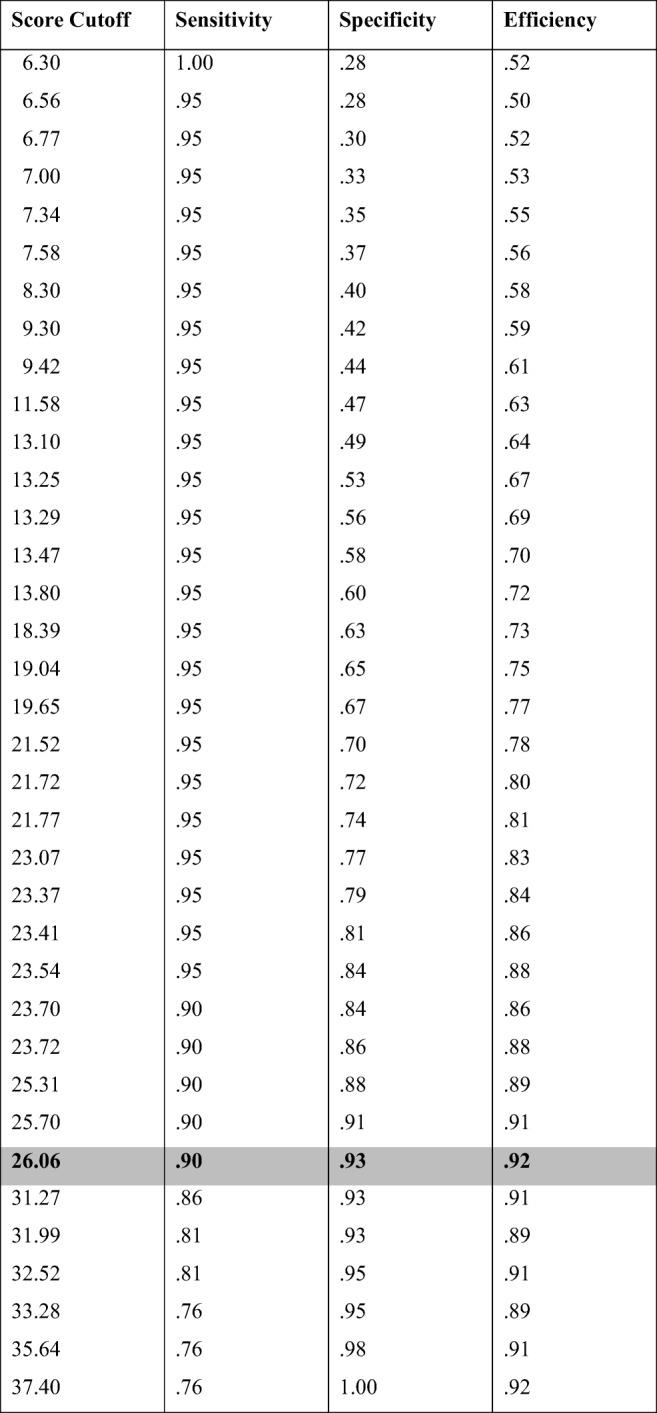
Sensitivity and specificity values for increasing the 5-Tastes cutoff scores, ranging from sensitivity = 1 to specificity = 1

The cutoff (26.00%) with maximum efficiency is highlighted in gray. Sensitivity represents the probability of detecting synesthesia in self-declared synesthetes, whereas specificity is the probability of correctly rejecting self-declared nonsynesthetes. Efficiency represents the proportion of that are classified according to self report

Unlike in Experiment 1, our test initially appeared to be sensitive to the distinction between associator and projector synesthetes. There was a significant difference between the groups in terms of consistency, *t*(19) = 2.19, *p* = .041, Cohen’s *d* = 0.98, with associators being more consistent (*N* = 10, *M* = 76.25, *SD* = 22.92) than projectors (*N* = 11, *M* = 52.46, *SD* = 26.44). However, when we explored this effect, it appeared to be carried by one outlier participant who classified herself as an projector but performed very poorly in our diagnostic of synesthesia (her consistency was 6% only). When this outlier was removed, there was no longer any significant difference across associators and projects, *t*(18) = – 1.77, *p* = .077, Cohen’s *d* = 0.84.

### Discussion

Our diagnostic test for LG synesthesia was able to separate synesthetes from nonsynesthetes not only group-wise, but also with a useful threshold cutoff with “excellent” predictive power. In our task, participants completed a self-report questionnaire for LG synesthesia and then an objective “test of consistency,” which required them to think of a food association for each of 30 words. Participants indicated their food associations using a pie chart of tastes, on which they could represent the relative contributions of sweet, sour, salty, bitter, and umami to the overall flavor of their food. Our dependent measure was an indicator of how consistently they made these food associations for any given word across a test and retest. This measure was a percentage derived from a mean *R*^2^ averaged across responses for the five tastes. This value represents how well food responses in the retest could be predicted from those given in the original test. In other words, it predicted how sweet, salty, bitter, sour, and umami any word would be, given how that same word had been rated previously. Synesthetes had a significantly higher consistency score than nonsynesthetes. Their average score was 70%, whereas the average for nonsynesthetes was far lower (13%). Furthermore, the test was so difficult for nonsynesthetes that they clustered together in this low band, and could therefore be easily distinguished from synesthetes around a threshold of just 26%.

Finally, we again found evidence that we had selected our target words well. As in Experiment 1, synesthetes experienced synesthetic foods on average for 80% of our words, and all of the words elicited taste associations. This is again considerably higher than has been found in other word lists that had not been so carefully planned in this regard (cf. < 60% in Ward et al., 2005).

## General discussion

Our aim has been to provide an objective, online test for the diagnosis of LG synesthesia. We successfully achieved our research aims of providing an online platform where LG synesthetes could be distinguished from nonsynesthetes in an automatic way with a threshold cutoff. LG synesthetes experience automatic food associations triggered by words and are traditionally recognized by the high consistency over time with which they match words to foods in repeated testing. Earlier studies using this “test of consistency” methodology had a number of drawbacks. Although the earlier tests were robust, they were not automated, were not available via any online platform, required human intervention from coders, were coded only subjectively, did not have well-designed lists of “highly tasty” words, and took several months to administer before a diagnosis could be reached. All of these features have been overcome in the tests we presented here. Our two tests each provided an automated online tool to separate groups of synesthetes from nonsynesthetes. Our first test also provided a “good” cutoff threshold for synesthesia, whereas our second test provided an “excellent” threshold. For this reason, we promote here our second test, the 5-Tastes pie-chart method, for future studies wishing to diagnose LG synesthesia. We have named this test the MULTISENSE Test for Lexical–Gustatory Synaesthesia.

In both our experiments, participants were given a clear definition of LG synesthesia with examples and were asked to self-report whether they experience LG synesthesia. In both experiments participants were shown 30 words once in a randomized list, and then again in a second, rerandomized block immediately afterward. In both experiments, participants were required to choose a food association for each word (e.g., to choose a food for the word “distance”), and we compared how consistently these foods were chosen across the first and second presentations of each word (i.e., at test and retest). In Experiment 1, food items were selected by participants from an onscreen food palette, which showed 87 subordinate foods (e.g., butter) under 11 superordinate food categories (e.g., fats). We also elicited the intensity of each word–food association. Synesthetes were significantly more consistent than controls in both of these measures. In Experiment 2, food items were described using an on-screen pie chart of five basic tastes. Participants manipulated the pie chart to show the relative contribution of each taste to the overall flavor of their associated food (e.g., the word “child” might be associated with the food lemon drops, which might then be rated on the pie chart as 50% sweet, 50% sour, 0% umami, 0% salty, and 0% bitter). For our dependent measure, we regressed each person’s tastes in the pie chart across the first and second presentations of each word (giving each person an *R*^2^ for sweetness, and *R*^2^ for sourness, etc.). We then averaged these five *R*^2^s to give each participant a mean *R*^2^ across their five tastes, and finally converted this value to a percentage for each participant. This final score was the dependent measure we promote here as our multisense score for LG synesthesia. We found that nonsynesthetes scored similarly to each other and very poorly in this measure, whereas synesthetes scored considerably higher. Indeed, a threshold of 26% would distinguish synesthetes from nonsynesthetes with excellent power, in terms of both sensitivity (including self-declared synesthetes) and specificity (correctly excluding nonsynesthetes). We therefore promote this as an automated online consistency test for LG synesthesia.

The discriminant power of our test was comparable to the most widely used online test for verifying grapheme–color synesthesia, in which people experience colors triggered by letters or numbers (Eagleman et al., 2007). Rothen, Seth, Witzel, and Ward (2013) examined the discriminant power of this test by applying ROC analyses as we have here, and they reported a possible AUC value of .92, comparable to the AUC of .95 observed here. Where this commonly used grapheme–color test was shown to have an 88% chance of classifying self-declared synesthetes as synesthetes and an 11% chance of classifying self-declared nonsynesthetes as synesthetes, our novel test for LG synesthesia passed 94% of self-declared synesthetes and 7% of controls in our sample with a 26% cutoff.

We chose to include no more than 30 words in our stimulus set, because our aim was to design a consistency test with a short completion time that could be used alongside other tasks in research studies and would be less prone to participant dropout, particularly in online studies. We felt that this number was appropriate because it is close to the numbers of inducers presented in other automated tests (e.g., tests for sequence–space synesthesia present either 7, 12, 10, or 29 inducers, and grapheme–color tests present 10, 26, or 36) that work well at verifying synesthesia. We chose words of high rather than low frequency because we were interested in creating a set of typical inducers that would elicit taste associations in as many synesthetes as possible, rather than a word set that would capture more atypical associations but would not reliably elicit associations in the majority of synesthetes.

Despite our efforts fine carefully selecting the stimuli, not every synesthete reported a taste association for all 30 words. This is perhaps unsurprising; synesthetes vary from one to another not only in the percentage of words that trigger tastes (e.g., see Ward et al., 2005), but also in the way their past experiences favor tastes for some words over others. For example, tastes are closely related to childhood diet (i.e., foods eaten often in childhood are more likely to become synesthetic tastes; Ward & Simner, 2003), and tastes can also be traced through phonological neighborhoods (e.g., “reach” tends to taste of peach; Simner & Haywood, 2009). Hence, if the foodstuff treacle, for example, featured in the childhood diet of one particular synesthete, this would increase the likelihood of a taste for our target word “reason” (which falls within the same phonological neighborhood as “treacle,” given the overlapping phoneme cluster /ri/). Another synesthete with different dietary experiences would be less likely to develop that pairing. In other words, whether or not a given word takes on a taste is the result of a complex interaction between diet and language, and it is therefore unsurprising that synesthetes differ in the number and nature of their associations. Nonetheless, we took great care to choose target words that are known to increase the likelihood of tastes overall (e.g., high-frequency words).

We will end our article with a brief discussion of the status of LG synesthesia as a “condition” that might be “diagnosed” with our test. But we wish to be clear that by using the word “diagnose,” we are not implying that LG synesthesia is an illness, and we are certainly not implying a need to cure it. The key issue here is that LG synesthesia manifests itself in many different ways for different LG synesthetes, and although many LG synesthetes experience no deficits from their experiences, a smaller number experience some problems—particularly if their tastes are projected, rather than associated, flavors. Some LG synesthetes have reported experiencing overwhelming or unpleasant flavor experiences (e.g., vomit, earwax), which are unwanted or distracting (e.g., when driving). These can sometimes lead to “sensory overload” in loud or busy environments, and have even led some synesthetes to fundamentally change key aspects of their professional or social life (switching jobs to quieter environments, or avoiding friends with unpleasant-tasting names). However, other people with synesthesia will show no negative impact whatsoever. The weight of this evidence suggests that we might consider synesthesia in two different ways—as either a “condition” (for those synesthetes with greater difficulties) or simply a “trait” (for those without)—and that our test could therefore either “diagnose” or simply “identify” it. Either way, a clear test for LG synesthesia is a much-needed addition to the science literature, and providing such a test has been our aim in the present article.

## Electronic supplementary material

ESM 1 (DOCX 31 kb)

## Footnotes

1 Earlier we clarified the technical difference between “taste” and “flavor,” but use the former word in our communication with participants because it is the most commonly used lay term.
