# Supplementary material for: The MULTISENSE Test of Lexical–Gustatory Synaesthesia: An automated online diagnostic
Source: Behav Res Methods. 2019 Jun 3;52(2):544–60. doi: 10.3758/s13428-019-01250-0 (PMC7148268; doi:10.3758/s13428-019-01250-0)
Supplement: Supplementary file 1 — (DOCX 31 kb) [file 13428_2019_1250_MOESM1_ESM.docx]

**SUPPLEMENTARY INFORMATION**

***Testing the usability of the two test interfaces***

To ensure that individuals would be able to report tastes consistently using our interface, we ran a pilot study in which we asked 9 participants without lexical-gustatory (LG) synaesthesia (3 males, Mean age = 33.33, SD = 8.38) to complete each of our tasks, but with one difference. As in our main studies, pilot subjects used our interface to specify foods from a category list (Experiment 1) or in terms of their 5 basic tastes (Experiment 2). But whereas subjects in our main study made their own decisions about which foods they were targeting (i.e., triggered synaesthetically from a word-list or by free association, for synaesthete and controls respectively), here we provided those foods a priori. So whereas a control in our main study might see the word “America” and free associate to hamburgers, here we provided the food itself. So for example we provided the word “hamburger” as a stimulus item, and the pilot subject was required to specify this food either by using our food-category list (as in Experiment 1) or our 5-tastes pie chart (as in Experiment 2). Our aim here was to ensure that foods could, indeed, correctly be specified consistently with our interfaces. Our stimuli list of foods was taken from true synaesthetic food descriptions given to us previously from LG synaesthetes in our earlier studies (e.g., in Simner & Ward, 2006). These foods are shown in Table S1 below. We choose foods to reflect a range of tastes, a range of ingredients and to vary in terms of how difficult they might be to categorise using the palette or pie chart.

**Figure Table S1:** Food items presented in the pilot study for the Food Categories test interface (left column) and 5-tastes task (right column) in alphabetical order.

| **Food Categories (Pilot Task)** | **5 Tastes (Pilot Task)** |
| --- | --- |
| Apricot yoghurt | Apple sauce |
| Black Liquorice | Aspirin |
| Boiled Cabbage with a piece of butter | Beef Gravy |
| Boiled hot-dogs | Black Liquorice |
| Cinnamon buns | Black Olives |
| Cold baked beans in tomato sauce | Boiled green beans with butter |
| Cream of mushroom soup, cooling off | Boiled onion in soup |
| Crispy bacon | Boiled potato |
| Digestive biscuit dipped in milk | Burnt toast |
| Fresh celery with some mayonnaise | Cheese toastie (white bread) |
| Fresh ripe cantaloupe | Chips with ketchup and cheese |
| Freshly brewed coffee with milk | Chocolate pudding |
| Fried soggy onions | Coriander |
| Green grapes in fruit cocktail | Fresh carrots |
| Green Olives | Fresh orange juice |
| Hamburger (no bun) with ketchup | Fresh pineapple |
| Homemade green pea soup | Fried crispy onions |
| Hummus with paprika | Kellogg's cornflakes |
| Mild peppermint | Macaroni and Cheese |
| Nougat filling in a box of chocolates | Mushroom risotto |
| Porridge when it cools down | Raspberry jelly |
| Rice crispy squares | Sauerkraut |
| Rubbing alcohol | Slightly boiled cabbage without butter |
| Salami | Snickers bar |
| Slightly burnt and dry scrambled eggs | Spaghetti Bolognese |
| Soap | Steak fairly well-done near the bone |
| The white sauce in lasagne | The coating from a red candied apple |
| Tinned tuna in brine | The cone from an ice-cream cone |
| Tobacco | Tinned Tomato Juice |
| Vegetable stock with noodles | Watermelon that's not crisp |

As in the main task, we presented each stimulus list twice, and randomised the order of the stimuli within each presentation. We calculated the consistency with which participants categorised the foods across the two presentations using the same methods described in the main methods section. To briefly remind the reader, for the Categories Task, we counted partial and complete category matches across the two repetitions and divided by the total number of responses that had a taste response. In this pilot, there were no trials in which participants pressed the ‘No-Taste’ button, and therefore the number of matches was divided by the number of trials (N = 30). For the 5 Tastes task, the consistency score was calculated by regressing responses collected during the first presentation of words against the responses from the second presentation for the same words, for each of the 5 tastes. The 5 resulting R Squared values were then averaged to produce one mean value, and then converted into a percentage.

On the Categories task, the mean consistency score was 87.04 (SD = 10.86). On the 5 Tastes task, the R^2^ consistency score was 66.00 (SD = 13.61). A comparison of this score and that of synaesthete and non-synaesthete control scores from the corresponding main task can be seen in Figure S1 (Food categories task; see Experiment 1) and in Figure S2 (5 Tastes task; see Experiment 2). The conclusions from our pilot study are that our subjects could not only use our testing information as required for them, but that their food-selections were consistent over time, as we might hope. And it is interesting to note that when pilot (non-synaesthetic) subjects are describing stimuli foods, they do so in a highly similar way to synaesthetes describing their *synaesthetic* foods.

**Figure S1:** Mean consistency scores from Experiment 1 for LG synaesthetes and non-synaesthetes, and from the pilot Experiment in which participants described food items using the categories interface. Error bars indicate 95% CIs.

**Figure S2:** Mean consistency scores from Experiment 2 for synaesthetes and non-synaesthetes, and from the pilot Experiment in which non-synaesthetes described food items using the 5 tastes interface. Error bars indicate 95% CIs
